# Modeling Neural Activity with Conditionally Linear Dynamical Systems

**Published:** 2025-02-25

**Authors:** Victor Geadah, Amin Nejatbakhsh, David Lipshutz, Jonathan W. Pillow, Alex H. Williams

**Affiliations:** 1 Program in Applied and Computational Mathematics, Princeton University, Princeton, NJ.; 2 Center for Computational Neuroscience, Flatiron Institute, New York City, NY.; 3 Department of Neuroscience, Baylor College of Medicine, Houston, TX.; 4 Princeton Neuroscience Institute, Princeton, NJ.; 5 Center for Neural Science, New York University, New York City, NY.

## Abstract

Neural population activity exhibits complex, non-linear dynamics, varying in time, over trials, and across experimental conditions. Here, we develop *Conditionally Linear Dynamical System* (CLDS) models as a general-purpose method to characterize these dynamics. These models use Gaussian Process (GP) priors to capture the nonlinear dependence of circuit dynamics on task and behavioral variables. Conditioned on these covariates, the data is modeled with linear dynamics. This allows for transparent interpretation and tractable Bayesian inference. We find that CLDS models can perform well even in severely data-limited regimes (e.g. one trial per condition) due to their Bayesian formulation and ability to share statistical power across nearby task conditions. In example applications, we apply CLDS to model thalamic neurons that nonlinearly encode heading direction and to model motor cortical neurons during a cued reaching task.

## 1. Introduction

A central problem in neuroscience is to capture how neural dynamics are affected by external sensory stimuli, task variables, and behavioral covariates. To address this, a longstanding line of research has focused on characterizing neural dynamics through recurrent neural networks (RNNs) and their probabilistic counterparts, state-space models (SSMs; for reviews, see Paninski et al. 2010; Duncker & Sahani 2021; Durstewitz et al. 2023).

Early work in this area utilized latent linear dynamical systems (LDS) with Gaussian observation noise. Although these assumptions are restrictive, they are beneficial in two respects. First, they simplify probabilistic inference by enabling Kalman smoothing and expectation maximization (EM)—two classical and highly effective methods (see e.g., Ghahramani & Hinton 1996). Second, they produce models that are mathematically tractable to analyze with well-established tools from linear systems theory (Kailath, 1980). Indeed, many influential results in theoretical neuroscience have come from purely linear models (e.g. Seung 1996; Goldman 2009; Murphy & Miller 2009).

In reality, most neural circuits do not behave like time-invariant linear systems. Thus, more recent work from the machine learning community has cataloged a variety of non-linear models for neural dynamics. Although these new models often predict held out neural data more accurately than LDS models, they are generally more difficult to fit and more difficult to understand. Thus, there has been a proliferation of competing architectures—e.g. RNNs (e.g. Pandarinath et al. 2018), transformers (e.g. Ye & Pandarinath 2021), and diffusion-based methods (Kapoor et al., 2024)—as well as competing training and probabilistic inference methods—e.g. generalized teacher forcing (Hess et al., 2023), amortized variational inference (e.g. Pandarinath et al. 2018), and sequential Monte Carlo (e.g. Pals et al. 2024). Choosing among these strategies and scientifically interpreting the outcomes is challenging.

Here we describe *Conditionally Linear Dynamical Systems* (CLDS) as a framework to jointly capture some of the benefits of the classical (i.e. linear) and contemporary (i.e. nonlinear) approaches to modeling neural data. CLDS models parametrize a collection of LDS models that vary smoothly as a function of an observed variable $u_{t}$ (e.g. measured sensory input or behavior at time t). Assuming the presence of $u_{t}$ is often a feature—not a bug—of this approach. Indeed, a common goal in neuroscience is to relate measured sensory or behavioral covariates to neural activity. Additional features of the CLDS framework include:

1. CLDS models are locally interpretable. Conditioned on $u_{t}$, the dynamics are linear and amenable to a number of classical analyses.
2. CLDS models are easy to fit (§2.3). If Gaussian noise is assumed, then exact latent variable inference (via Kalman smoothing) and fast optimization (via closed-form EM) is possible. Under more realistic noise models (e.g. Poisson), the posterior over latent state trajectories is still log concave and amenable to relatively fast and simple inference routines.
3. CLDS models are expressive. As $u_{t}$ changes, the parameters of the linear system are allowed to change *non-linearly*. Thus, CLDS can model complex dynamical structures such as ring attractors (§3.2), that are impossible for a vanilla LDS to capture.
4. CLDS models are data efficient. To the extent that LDS parameters change smoothly as a function of $u_{t}$, we can the recover the parameters of the dynamical system with very few trials per condition (§3.4). In fact, CLDS models can interpolate to make accurate predictions on entirely unseen conditions.

Finally, CLDS models have connections to several existing methods (§4). For example, they can be viewed as a dynamical extension of a Wishart process model (Nejatbakhsh et al., 2023) and an extension of Gaussian Process Factor Analysis (GPFA; Yu et al. 2009) with a learnable kernel and readout function that can vary across time and conditions. They are also similar to various forms of switching linear dynamical systems models (Petreska et al., 2011; Nassar et al., 2019; Hu et al., 2024). The key difference is that the “switching” in CLDS models is governed by an observed covariate vector, $u_{t}$, rather than by a discrete latent process. This makes inference in the CLDS model much more straightforward, albeit at the price of not being fully unsupervised.

## 2. Methods

### Notation

We use $f(\cdot )∼GP^{N}(m(\cdot ),k(\cdot ,\cdot ))$ with mean $m:X\to {\mathbb{R}}^{N}$ and kernel k:X×X→ℝ, to denote samples $f:X\to {\mathbb{R}}^{N}$ obtained from stacking independent Gaussian processes into an N-dimensional vector.

### 2.1. Conditionally Linear Dynamical Systems

**Figure 1.**
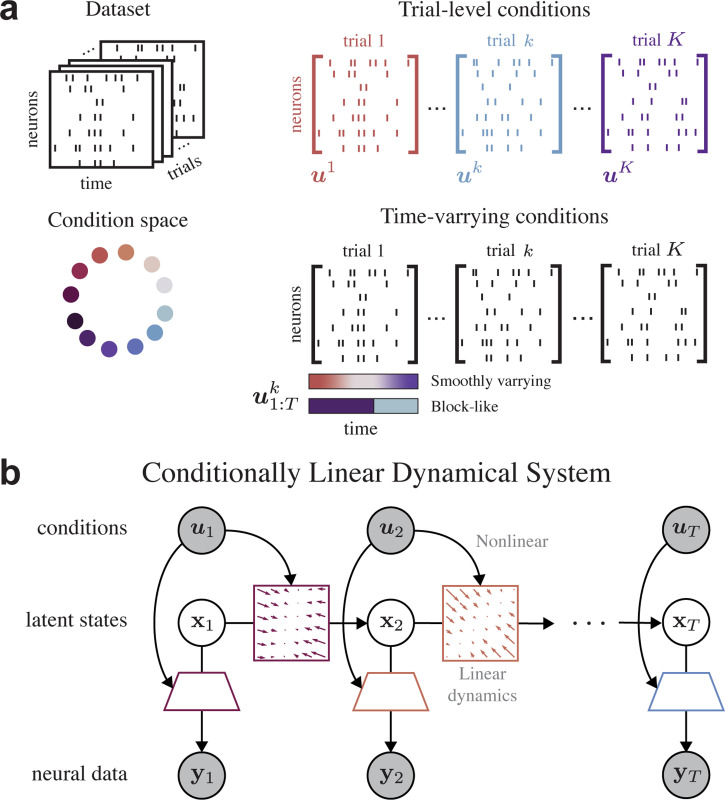
(**a**) Neural dataset consisting of spike trains collected over multiple trials, along with corresponding experimental conditions. (**b**) Conditionally Linear Dynamical Systems are *linear* in state-space dynamics and capture *nonlinear* dependencies over conditions. Shaded nodes are observed, clear nodes are latent.

Consider an experiment with N neurons recorded over K trials of length T. Our dataset consists of the recorded neural trajectories ${\left\{y_{1:T}^{k}\right\}}_{k=1}^{K}$, with $y_{t}\in {\mathbb{R}}^{N}$ at time-step t∈{1,…,T}, along with the corresponding experimental conditions ${\left\{u_{1:T}^{k}\right\}}_{k=1}^{K}$, with $u_{t}$ in the condition space U. By experimental conditions, we refer to available neural data covariates, either experimentally set or collected as measurements. These conditions, see Fig. 1a, can vary over time or remain constant (compare §3.3 vs. §3.4). They can also be a step function over time (e.g. animal moving vs. not moving), resulting in a switching-like mechanism between different dynamics.

We model response $y_{t}$ as emissions from a latent time-varying linear dynamical system in $x_{t}\in X\subseteq {\mathbb{R}}^{D}$, with the dynamics governed by the conditions $u_{t}$. Specifically,

(1a) $$x_{t+1}=A\left(u_{t}\right)x_{t}+b\left(u_{t}\right)+\epsilon_{t}$$

(1b) $$y_{t}=C\left(u_{t}\right)x_{t}+d\left(u_{t}\right)+\omega_{t}$$

evolving with time steps t∈{1,…,T} from initial condition $x_{1}∼N\left(x_{1};m\left(u_{1}\right),Q_{1}\right)$, and where $\epsilon_{t}∼N(0,Q)$ and $\omega_{t}∼N(0,R)$ are sources of noise, both sampled i.i.d. over time. We assume that the latent variables $x_{t}$ follow smooth dynamics defined by time-varying linear matrices $A(u)\in {\mathbb{R}}^{D\times D}$ from initial mean $m\left(u_{1}\right)\in {\mathbb{R}}^{D}$, with bias terms $b(u)\in {\mathbb{R}}^{D},d(u)\in {\mathbb{R}}^{N}$, and emissions governed by $C(u)\in {\mathbb{R}}^{N\times D}$. See graphical depiction in Fig. 1b.

Conditions are typically treated as additive *inputs*, influencing the dynamics in (1a) via additive terms of the form $Bu_{t}$ for a linear encoding matrix $B\in {\mathbb{R}}^{D\times |U|}$. Instead, the system in (1) parameterizes a *family of linear* systems indexed by a continuous time-varying variable, $u_{t}$, which is observed. In fact, the system in (1) can be thought of as the linearization in $x_{t}$ of a fully nonlinear system in $x_{t}$ and $u_{t}$, under additive noise—we explore this relationship in Appendix §A.2. The mapping of experimental conditions onto linear dynamics, u↦{A(u),b(u),C(u),d(u),m(u)}, is allowed to be nonlinear and learnable. Specifically, we place an approximate Gaussian Process (GP) prior on each entry of the parameters through a finite expansion of basis functions, leveraging regular Fourier feature approximations (Hensman et al., 2018). For any M∈{A,b,C,d,m}, we consider a prior over each i, j-th entry of the form:

(1c) $$M_{ij}(u)=\sum_{\ell =1}^{L}{\text{w}}_{\ell }^{\left(ij\right)}\phi_{\ell }(u),\;{\text{w}}_{\ell }^{\left(ij\right)}\overset{iid}{∼}N(0,1),$$

truncated at L∈ℕ basis functions. Intuitively, each basis function $\phi_{\ell }:U\to \mathbb{R}$ is fixed, and the randomness in the prior purely comes from the weights, ${\text{w}}_{\ell }^{\left(ij\right)}$, which are drawn from a standard normal distribution. When constructed appropriately, the prior in equation (1c) converges to a non-parametric Gaussian process in the limit that L→∞. In our experiments, the basis functions ${\left\{\phi_{\ell }\right\}}_{\ell =1}^{L}$ are chosen and scaled as to approximate a GP prior of the form $M_{ij}(\cdot )∼GP\left(0,k_{u}\right)$ for the squared exponential kernel $k_{u}$ with variance $\sigma^{2}$ and length-scale κ (Borovitskiy et al., 2020).

We denote F={A,b,C,d,m} as the set of random functions, and analogously the parameter set F(u)={A(u),b(u),C(u),d(u),m(u)} for any experimental condition u∈U. The model distribution

(2) $$p\left(y_{1:T},x_{1:T}|A,b,C,m,u_{1:T}\right)=\;\;p\left(y_{1:T},x_{1:T}|F,u_{1:T}\right)$$

describes a time-varying latent Linear Dynamical System, conditioned on a parameter sequence set at experimental conditions. Therefore, we refer to the model in equations (1) as a *Conditionally Linear Dynamical System* (CLDS). Our CLDS implementation is available at: https://github.com/neurostatslab/clds

### 2.2. CLDS modeling choices

Practitioners can adapt a CLDS model in several ways to suit different applications and modeling assumptions. First, the GP prior can be tuned to trade off model expressivity for interpretability and learnability. In one extreme, as we let κ→0, the LDS parameters change rapidly, nonlinearly as a function of u and become independent per u. In the other extreme, if one takes κ→∞, then the LDS parameters become constant (do not change as a function of u) and we recover a time-invariant LDS model with autonomous dynamics. In this regime, we could also modify the GP prior over b(⋅) to follow a linear kernel, $k\left(u,u^{\prime }\right)=u^{⊤}u^{\prime }$, resulting in time-invariant LDS with additive dependence on $u_{t}$. Thus, CLDS models capture classical linear models as a special case. Moreover, the model’s prior can be tuned to capture progressively nonlinear dynamics.

A second source of flexibility is the encoding of experimental covariates, u. Recall our notation from section 2.1, that $u_{t}^{k}$ represents experimental covariates at time t∈{1,…,T} and trial k∈{1,…,K}. A simple, and broadly applicable, modeling approach would be to set $u_{t}^{k}=t$. This achieves a time-varying LDS model in which the GP prior encodes smoothness over time. This is similar in concept to fitting an linear model to data over a sliding time window (see e.g. Costa et al. 2019; Galgali et al. 2023). However, the CLDS formulation of this idea is fully probabilistic, which has several advantages. For example, we will see that one can use a single pass of Kalman smoothing to infer the distribution over the latent state trajectory, $x_{1:T}^{k}$, within each trial. It is comparatively non-trivial to average latent state trajectories across multiple LDS models that are independently fit to data in overlapping time windows.

In section 3, we demonstrate more sophisticated examples where $u_{t}^{k}$ is specified to track a continuously measured behavioral variable (e.g. heading direction or position of an animal) or follow a stepping or ramping function aligned to discrete task events (e.g. a sensory “go cue” or movement onset). Section 4 discusses further connections between CLDS models and existing state space models.

### 2.3. Inference

As mentioned earlier, the conditional distribution in eq. (2) has the advantage of describing a latent linear dynamical system (LDS), or linear Gaussian state-space model. As such, we can benefit from analytic tools like Kalman filtering to compute the filtering distributions $p\left(x_{t}|y_{1:t},F,u_{1:T}\right)$ and marginal log-likelihood $p\left(y_{1:T}|F,u_{1:T}\right)$, and Kalman smoothing to compute the smoothing posterior $p(x_{1:T}|y_{1:T},F,u_{1:T})$. We focus on performing maximum-a-posteriori (MAP) inference for these parameters. In principle, it would be a straightforward extension to use variational inference or Markov Chain Monte Carlo to approximate the full posterior over these parameters.

#### Conditionally Linear Regression

As a stepping stone towards our goal of performing MAP inference for {A,b,C,d,m}, consider the model

(3a) $$y_{n}=M\left(u_{n}\right)x_{n}+\epsilon_{n},\;\epsilon_{n}∼N(0,\text{Σ})$$

(3b) $$M(\cdot )∼GP^{D_{1}\times D_{2}}\left(0,k_{u}\right),$$

given data $y_{n}\in {\mathbb{R}}^{D_{1}}$, regressors $x_{n}\in {\mathbb{R}}^{D_{2}}$, conditions $u_{n}\in U$, repeats n∈{1,…,N}, noise covariance Σ≻0, and with an approximate GP prior on M parametrized as in (1c). We refer to the model (3) as *conditionally linear regression*, and our goal is to perform MAP inference for the weights ${\left\{w_{k}^{\left(ij\right)}\right\}}_{i,j,k}$ for M.

Our parameterization in eq. (1c) implies that each entry is a dot product, $M_{ij}(\cdot )=w^{(ij)⊤}\phi (\cdot )$, where $\phi (\cdot )={\left(\phi_{1}(\cdot ),\ldots ,\phi_{L}(\cdot )\right)}^{⊤}\in {\mathbb{R}}^{L}$ is our vector of basis-functions evaluations. Therefore,

(4) $$M(u)=W^{⊤}\left(\phi (u)⊗I_{D_{2}}\right)$$

(5) $$M(u)X=W^{⊤}(\phi (u)⊗X),$$

for u∈U and for any vector or matrix $X\in {\mathbb{R}}^{D_{2},\ldots }$ of appropriate dimension, with “⊗” the Kronecker product, and with our weights aggregated into the matrix

(6) $$W_{j+\ell ,i}:=w_{\ell }^{\left(ij\right)},\;W\in {\mathbb{R}}^{D_{2}L\times D_{1}}.$$

With this, we can rewrite our regression problem as

(7) $$y_{n}=M\left(u_{n}\right)x_{n}+\epsilon_{n}=W^{⊤}z_{n}+\epsilon_{n}$$

with $z_{n}:=\phi \left(u_{n}\right)⊗x_{n}\in {\mathbb{R}}^{D_{2}L}$. Thus, we have reformulated our original model into Bayesian linear regression in an expanded feature space. Namely, the MAP estimate of the weights, $W_{\text{MAP}}$, is given by

(8) $$\underset{W}{\arg\max}\;\log p\left(y_{1:N}|W,x_{1:N},u_{1:N}\right)+\log p(W)$$

which is a linear regression problem with regularization from the prior $\log p(W)=-\frac{1}{2}∥W{∥}_{F}^{2}$ (up to an additive constant). We can analytically solve for the solution (derivations in Appendix §A.1.1), which yields that $W_{\text{MAP}}$ is the solution to the Sylvester equation

(9) $$Z^{⊤}ZW+W\text{Σ}=Z^{⊤}Y$$

with $Z\in {\mathbb{R}}^{N\times D_{2}L}$ our matrix obtained by stacking ${\left\{z_{n}\right\}}_{n=1}^{N}$, and similarly for $Y\in {\mathbb{R}}^{N\times D_{1}}$. We see that if $\text{Σ}=\sigma^{2}I_{D_{1}}$ for some σ>0 then we obtain back the familiar looking penalized least squares estimate $W_{\text{MAP}}={\left(Z^{⊤}Z+\sigma^{2}I_{D_{1}}\right)}^{-1}Z^{⊤}Y$.

#### Expectation Maximization

We can leverage the above to perform MAP inference for {A,b,C,d,m} with the *Expectation-Maximization* (EM) algorithm (Dempster et al., 1977; Ghahramani & Hinton, 1996). In the E-step we obtain estimates of the moments of the latents with Kalman-smoothing, which then place us in a setting akin to eq. (3) with sufficient statistics as data and regressors. We can then perform closed-form M-steps with our updates in eq. (9). We provide in Appendix §A.1.2 an example of the associated derivations with these E- and M-steps for the joint update for A(⋅) and b(⋅). The resulting EM algorithm has several advantages: (1) all E- and M-steps are analytic, (2) the E-step provides us with exact (penalized) marginal log-likelihood calculations, and (3) the algorithm gives monotonic gradient ascent guarantees of the marginal log-likelihood (resp. log posterior) objective.

We initialize the EM algorithm at samples from our GP priors for the parameters. With the EM algorithm we also learn the covariance parameters $\left\{Q_{1},Q,R\right\}$. Finally, the hyper-parameters {L,κ,σ} from the GP priors are determined through performance on held-out test sets from 80/20 trial splits on all experiments.

#### Extensions to non-Gaussian likelihoods

The closed form EM updates are only applicable when the distribution of $y_{t}$ conditioned on $x_{t}$ and $u_{t}$ is Gaussian. This assumption, stated in eq. (1), is common existing methods (e.g. Yu et al. 2009; Williams et al. 2018). However, alternative models are likely a better fit to many neural datasets. For spike count data, past work has utilized Poisson (e.g. Macke et al. 2011) and COM-Poisson (Stevenson, 2016) likelihoods.

Although we assume a Gaussian likelihood in our experiments in §3, we note that inference in CLDS models remains tractable whenever $p\left(y_{t}|W,x_{t},u_{t}\right)$ is log concave. This condition satisfied by most likelihood models of interest (e.g. Poisson). Indeed, conditioned on $u_{1:T}$, the log posterior density associated with $x_{1:T}$ is equal to a sum of concave terms up to an additive constant (Paninski et al., 2010). Thus, we can use standard optimization routines to identify a MAP estimate of $x_{1:T}$ efficiently with theoretical guarantees. This can then be used to implement an approximate EM algorithm (Macke et al., 2011).

## 3. Experiments

### 3.1. Setup

#### Metrics

For a given trajectory $\left\{y_{1:T},u_{1:T}\right\}$, we denote as *data reconstruction* the mean emission $E\left[y_{1:T}|{\hat{x}}_{1:T},F\left(u_{1:T}\right)\right]=\left(C\left(u_{1}\right){\hat{x}}_{1},\ldots ,C\left(u_{T}\right){\hat{x}}_{T}\right)$ from a the posterior mode ${\hat{x}}_{1:T}$, computed with Kalman smoothing, given the observations $y_{1:T}$ and parameters $F\left(u_{1:T}\right)$. As our primary metric, we use *co-smoothing* (Pei et al., 2021) to evaluate the ability of models to predict held-out single-neuron activity. Specifically, for the top 5 neurons with highest variance from the test set, we compute the coefficient of determination $R^{2}$ between the true and reconstructed single-neuron firing rate, obtained by performing data reconstruction using only the other neurons.

#### Composite dynamics

The latent dynamical system (1a) depends on the condition $u_{t}$, which can make visualizations challenging. Building on the idea that CLDS models decompose a nonlinear dynamical system into linearizations governed by u (see Appendix §A.2), we aim to approximate the general nonlinear system by marginalizing out $u_{t}$, conditioned on $x_{t}$. That is,

(10) $$x_{t+1}=g\left(x_{t}\right)+\epsilon_{t}\;\;:=E_{p\left(u|x_{t}\right)}\left[A(u)x_{t}+b(u)\right]+\epsilon_{t},$$

which we define as the *composite dynamical system*. Intuitively, we expect this to provide a good approximation to the underlying nonlinear dynamics when $u_{t}$ and $x_{t}$ tightly co-determine each other—i.e., when the encoding $p\left(x_{t}|u_{t}\right)$ and decoding $p\left(u_{t}|x_{t}\right)$ conditional distributions have low variance (see Appendix §A.2.2). In practice, we estimate the expectations in (10) by computing the empirical average over u per binned $x_{n}$, obtained by pooling the $u_{n}$ associated with the posterior mode ${\hat{x}}_{n}$ given a trajectory $\left\{y_{1:T},u_{1:T}\right\}$.

#### Model baselines

For model comparison, we use as baselines (1) a standard **LDS** model with additive inputs of the form $Bu_{t}$ in the latent dynamics, and (2) the **LFADS** (Pandarinath et al., 2018) model with controller inferred-inputs, with Gaussian observation model to fit directly to the firing rates. See Appendix §B.1 for implementation details.

### 3.2. Synthetic Head-Direction Ring Attractor

**Figure 2.**
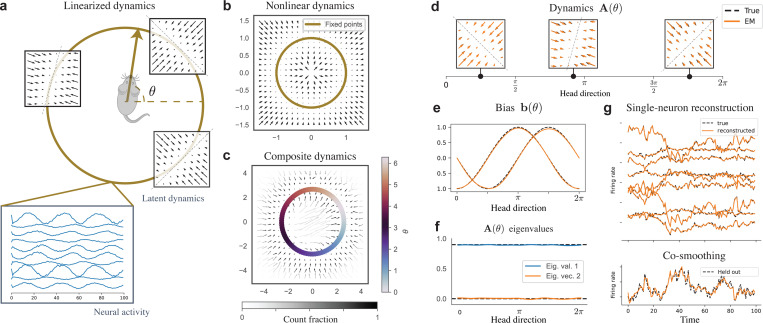
Head direction synthetic experiment. (**a**) Schematic of latent dynamics and neural activity about θ∈[0,2π), the mouse HD, serving as conditions u=θ in this task. (**b**) True nonlinear flow-field corresponding to the schematic in **a**, computed considering $p(\theta |x)=\delta_{∠x}(\theta )$. (**c**) Recovered composite dynamics by CLDS, see text for computation details. Grey scale indicates $x_{t}$ occupancy. The model fixed points (colored) as a function of θ form a perfect ring, overlapping with the true fixed points. (**d**-**e**) Parameter recovery for the dynamics matrix A(θ) (**d**) and the bias b(θ) (**e**) as functions of head direction θ. (**f**) Recovered eigenvalues of A(θ) as a function of θ, true in dashed. (**g**) Co-smoothing reconstruction from the test-set. The firing rate of one neuron is held-out (bottom) while the rest (top) is observed, and we reconstruct accurately the single-neuron firing rates for both the held-in (top) and held-out (bottom) neurons.

We start by considering a synthetic experiment of head direction neural dynamics. We conceptualize latent dynamics that capture the head direction (HD) of the animal, with attractor dynamics about a HD-dependent fixed point—see schematic in Fig. 2a. This synthetic experiment is designed to represent a nonlinear system decomposed as linear systems, per HD serving as the condition. We plot in Fig. 2b what the resulting, “composite dynamics” (see §3.1), nonlinear flow-field would be, assuming the latent state encodes the head direction exactly. The generative dynamics are an instance of a CLDS model by construction, so this synthetic example allows us to explore recovery performance.

Concretely, let $\theta_{t}\in [0,2\pi )$ denote heading direction at time step t, which we treat as our conditions $u_{t}:=\theta_{t}$. To build a ring attractor, we parametrize two orthogonal unit vectors

(11) $$e_{1}(\theta )=\left[\begin{array}{c} \cos(\theta )\\ \sin(\theta ) \end{array}\right],\;e_{2}(\theta )=\left[\begin{array}{c} -\sin(\theta )\\ \cos(\theta ) \end{array}\right],$$

that describe the position on the ring and the tangent vector respectively. We design (i.e. impose) that the system converges to a stable fixed point at $e_{1}(\theta )$, and at head direction θ we approximately integrate speed input along the subspace spanned by $e_{2}(\theta )$. To do this, we define A(θ) to be a rank-one matrix that defines a leaky line attractor, with attracting (i.e. contracting) dynamics along the orthogonal $e_{1}(\theta )$. For a hyperparameter 0<ϵ<1 define:

(12) $$A(\theta ):=(1-\epsilon )e_{2}(\theta )e_{2}{\left(\theta \right)}^{⊤},\;b(\theta ):=e_{1}(\theta ).$$

Completing the model description, we assume that the firing rate of individual neurons is given by a linear readout. For neuron i, the firing rate at time t is:

(13) $$y_{t,i}=C_{i,:}{\left(\theta_{t}\right)}^{⊤}x_{t}+\omega_{t}$$

where $C_{i,:}$ is sinusoidal bump tuning curve function (see App. §B.2). Note that we set $d\left(u_{t}\right)=0$. Finally, we sampled trials of length T=100 with M=10 neurons, generating the evolution of the heading direction as a random walk, $\theta_{t}∼N\left(\theta_{t-1},0.5^{2}\right)$, and initialize at the origin $x_{1}∼N(0,1)$.

We report our recovery results in Fig. 2, fixing the decoding matrix C(⋅) to a known value as to avoid non-identifiability considerations. We refer to Appendix §B.2 for recovery plots of C when fitted. First, we observed that we can generally recover the nonlinear flow-field, plotting in Fig. 2c the composite dynamics obtained from the posterior trajectories. This paints an activity-based portrait of the dynamics, and our ability to accurately estimate the flow-field around a given point under this method depends on how many posterior samples pass by it. We thus indicate, by gray-scale shading the flow-field arrows, the fraction of posterior samples pooled per bin as a fraction of the highest bin count.

Second, for a more parameter-based account of the recovery, we plot in Fig. 2d-e the varying biases b(θ) and dynamics matrices A(θ) as functions of the head direction θ—we recovered with high-fidelity the true parameters. This recovery translated into the properties of the dynamics such as the recovered eigenvalues of A(θ) in Fig. 2f. Finally, we observed that the test data single-neuron reconstruction (Fig. 2g) recovers the true observations, and the model was able to accurately $\left(R^{2}=0.86\right)$ reconstruct a held out neuron from this test-set through co-smoothing.

### 3.3. Mouse Head-Direction Circuit Dynamics

**Figure 3.**
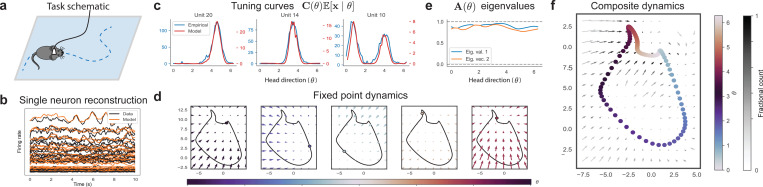
(**a**) Schematic of mouse foraging in an open environment. We have access to $\theta_{t}\in [0,2\pi )$ the mouse HD in time t, which we use as conditions $u_{t}$ just like Fig. 2. (**b**) Model reconstruction on the whole dataset recovers the true data. We plot single-neuron traces, averaged over 10s trials. (**c**) Model tuning curves over head direction θ, obtained as C(θ)E[x∣θ], recover the empirical tuning curves. Plotted for the top three units in firing rate norm. (**d**) Dynamics around each fixed point in x-state space as a function of head direction θ, with the solid-line representing the complete set of fixed points. (**e**) Eigenvalues and angles of eigenvectors of A(θ) as a function of θ. (**f**) Composite dynamics in $x_{t}$-space, with overlaid colored model fixed points as a function of θ.

Next, we turned to the analysis of antero-dorsal thalamic nucleus (ADn) recordings from Peyrache et al. (2015) of the mouse HD system in mice foraging in an open environment (Fig. 3a). We considered neural activity from the “wake” period, binned in 50ms time-bins, then processed to firing rates and separated into 10s trials. As with the synthetic experiment of the previous section, we treat the recorded head-direction $\theta_{t}\in [0,2\pi )$ as conditions $u_{t}=\theta_{t}$.

We recovered single-neuron firing rates with high accuracy (Fig. 3b) through data reconstruction. We further validated our fit by computing the empirical tuning curves, which our model recovered almost exactly (Fig. 3c). The model tuning curves are given by

(14) $$E\left[y_{i}|\theta \right]=E\left[E\left[y_{i}|x,\theta \right]\right]=C_{i,:}{\left(\theta \right)}^{⊤}E[x|\theta ],$$

which follows from the law of total expectation. The later quantity E[x∣θ] represents the expected encoding of the conditions θ, which we estimate by averaging posterior trajectories, obtained with Kalman smoothing, over (binned) θ given corresponding firing rates.

Finally, we analyzed the learned latent dynamics. Like the synthetic example, we identified a ring attractor structure (Fig. 3d). Now, unlike the synthetic example, we observed that this ring attractor is composed of HD-dependent fixed points as opposed to line attractors, as per the eigenvalues of A(θ) in Fig. 3e.

### 3.4. Macaque Center-Out Reaching Task

**Figure 4.**
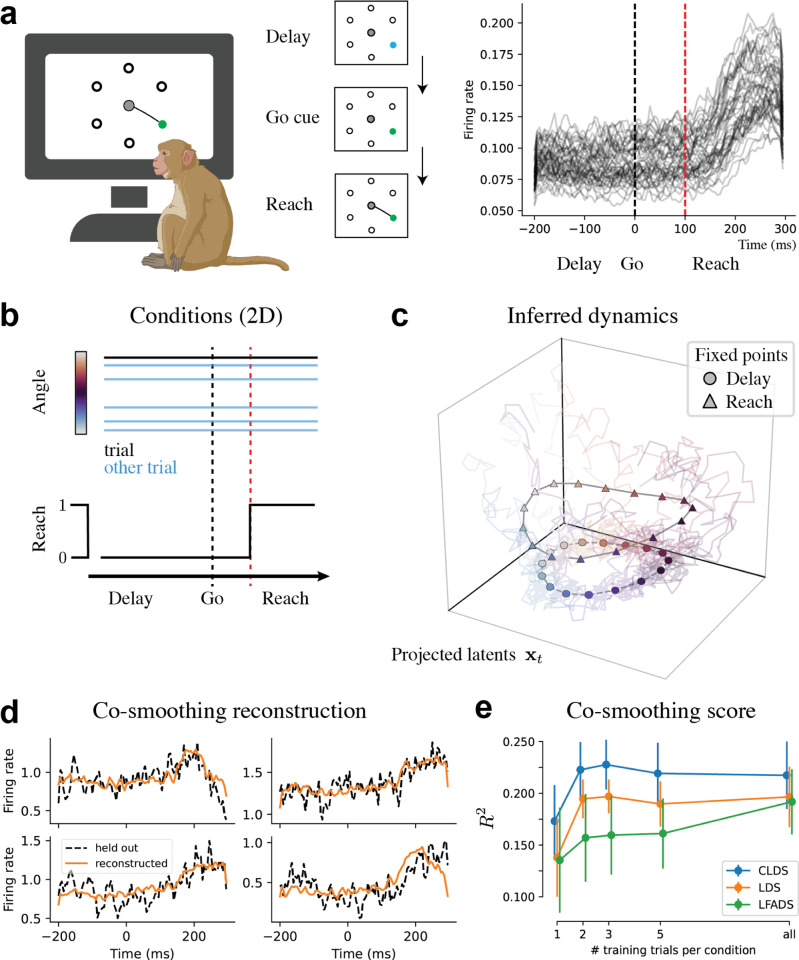
Macaque reaching experiment. (**a**) Task schematic (left) and population-averaged firing rates per trial (right). (**b**) 2D conditions, with trial orientation θ, and reach variable $z_{t}\in \{0,1\}$ switching at ramp onset. (**c**) 3D projection of the 5 dimensional latents used, projected as to align best with condition decoding. We show the model fixed-points per reach angle θ and reach condition z, plotted over posterior mean trajectories per trial. (**d**) Co-smoothing reconstruction of single held-out neurons from the test-set. (**e**) Co-smoothing $R^{2}$ per model as a function of the number of trials used per reach angle during training. Error bars indicated std. around the mean over 5 random initialization seeds.

Finally, we analyzed neural recordings of dorsal premotor cortex (PMd) in macaques performing center-out reaching task (Fig. 4a) from N. Even-Chen, B. Sheffer et al. (2019). In contrast to the previous experimental conditions, we consider here two-dimensional conditions $u_{t}^{k}=\left(\theta^{k},z_{t}\right)$, where $\theta^{k}\in [0,2\pi )$ is the instructed reach angle, constant per trial k, and $z_{t}\in \{0,1\}$ indicates the task reach condition (see Fig. 4b) set at 0 during the delay and 1 at 100ms past the go-cue, at the onset of the movement-related firing rate ramp Fig. 4a-(right). Discrete-valued conditions, such as the reach onset $z_{t}\in \{0,1\}$, are considered as supported on a continuous interval. The correlation between such discrete points is determined by the length-scale parameter κ, which we’ve set to κ=0.5 from a hyperparameter search. More details on hyperparameters and data-preprocessing can be found in Appendix §B. Finally, we use a fixed emission matrix C and let the latent dynamics capture the dependency on experimental conditions through A(u) and b(u).

We found the latent dynamics to encode the conditions through attracting fixed-points during both the delay and reach periods. We show in Fig. 4c the projection of the D=5 latent dimensions along the 3-dimensional subspace most aligned (i.e. best decoding) with the experimental conditions, following similar analyses from (N. Even-Chen, B. Sheffer et al., 2019)—we observed clear aligned rings of fixed points from delay to reach. In CLDS models, we obtain the fixed points by simply solving for $x^{*}(u)$ satisfying $\left(I-A(u))x^{*}=b(u\right)$ for any u, in contrast to numerical fixed-point methods usually employed (Sussillo & Barak, 2013).

We performed co-smoothing (see §3.1) to evaluate the model. We recovered with good accuracy single held-out neurons from the validation set excluded from training (Fig. 4d). We then compared the performance of the CLDS against the LDS and LFADS models, exploring further how each fares in low-data regimes. We report in Fig. 4e the co-smoothing $R^{2}$ per model, computed as a function of the number of trials used in each reach-angle $\theta^{k}$, averaged over 5 random seeds. We found that the CLDS outperformed both models consistently, with the highest difference at 1 training trial per condition. While the LFADS model showed progressively better performance that did not plateau yet, it nonetheless underperformed in these low data regimes.

## 4. Related work

### Wishart Process Models

CLDS models capture the dependence of neural responses $y_{1:T}$ on continuous experimental conditions $u_{1:T}$. Nejatbakhsh et al. (2023) investigated a very similar problem, focusing on single-trial responses $y_{k}$ to continuous experimental conditions $u_{k}\in U$. They use a conditional Gaussian model for responses $y_{k}$ given conditions $u_{k}$

(15) $$y_{k}|u_{k}∼N\left(y;\mu \left(u_{k}\right),\text{Σ}\left(u_{k}\right)\right)$$

and they place Gaussian process and Wishart process (Wilson & Ghahramani, 2011) priors on the mean and covariance functions. Concretely, they posit that

(16) $$\mu (\cdot )∼GP^{M}\left(0,k_{\mu }\right),$$

(17) $$\text{Σ}(u)=U(u)U{\left(u\right)}^{⊤}+\text{Λ}(u)$$

with $U(\cdot )∼GP^{M\times p}\left(0,k_{\text{Σ}}\right)$ and $\text{Λ}(\cdot )∼GP^{M}\left(0,k_{\text{Λ}}\right)$ for chosen kernel functions $\left\{k_{\mu },k_{\text{Σ}},k_{\text{Λ}}\right\}$. The hyper-parameter p∈ℕ determines the low-rank structure of Σ.

For a single time stepm, T=1, t=1, our system in (1) reads

$$\begin{array}{c} p\left(x_{1}|u_{1}\right)=N\left(x_{1};m\left(u_{1}\right),Q_{1}\right)\\ p\left(y_{1}|x_{1},u_{1}\right)=N\left(y_{1};C\left(u_{1}\right)x_{1}+d\left(u_{1}\right),R\right), \end{array}$$

Assuming a degenerate prior that $m\left(u_{1}\right)=0$, the marginal distribution of $y_{1}$ conditioned on $u_{1}$ equals

(17) $$N\left(d\left(u_{1}\right),C\left(u_{1}\right)Q_{1}C{\left(u_{1}\right)}^{⊤}+R\right).$$

which can be compared with (15) and (16). We observe that the models are essentially equivalent with μ(u)=d(u), and with the CLDS emission matrix C(u) serving as the Wishart process prior decomposition matrix U(u), right-scaled by $Q_{1}^{1/2}\in {\mathbb{R}}^{D\times D}$. This makes the parameter p=D now bear meaning as the dimensionality of the latents $x\in {\mathbb{R}}^{D}$. Thus, we can view CLDS models as a direct extension of Wishart process models that capture condition-dependent dynamics across multiple time steps.

### Markovian GPs

Latent GP models (Lawrence, 2007; Wang et al., 2005), such as the foundational model of GPFA by Yu et al. (2009), are widely used in neuroscience. Here we show that one can view MAP inference in a CLDS model as optimizing a kernel that defines a latent GP prior. While GPFA is not a dynamical system model, Yu et al. (2009), as well as Turner & Sahani (2007), detail how the the stationary dynamics of an AR-1 process (i.e. linear dynamical system) can be expressed as draws from a GP. More generally, all stationary, real-valued, and finitely differentiable GPs admit a representation in terms of linear state space models (Dowling et al., 2021; 2023). The main departure with our work is that we additionally place a GP prior on the *parameters* (coefficients) of an LDS, allowing the dynamics to vary across conditions and across time. For a fixed set of LDS parameters, F, and experimental covariates, $u_{1:T}$, the distribution of latent states in a CLDS are jointly Gaussian. Thus, a set of LDS parameters induces a (generally non-stationary) GP prior on the latent trajectories. In this view, the GP prior we place over the parameters of the LDS can be seen as a hyperprior over the latent dynamical process.

### Switching Dynamical Systems

A second class of relevant models generalizing the LDS are *Switching LDS* models (SLDS; Murphy 1998; Pavlovic et al. 2000; Petreska et al. 2011). SLDS models consist of a discrete latent state ${\text{z}}_{t}$ with Markov chain dynamics dictating the dynamics matrix $A^{\left({\text{z}}_{t}\right)}$. This switching behavior can be mimicked in our setting if the condition space is discrete (see, e.g., §3.4). We can take the relationship a step further by embedding the discrete process in the continuous dynamics parameter space of $A^{\left({\text{z}}_{t}\right)}\in {\mathbb{R}}^{D\times D}$. Under this lens and in a similar line of thinking as with Markovian GPs, we show in Appendix §A.3 how a one-dimensional SLDS model with latent dynamics

$$p\left({\text{z}}_{t+1}=i|{\text{z}}_{t}=j\right)=P_{ij},\;{\text{x}}_{t+1}=a^{\left({\text{z}}_{t}\right)}{\text{x}}_{t}+\epsilon_{t}$$

is equivalent, up to the first two moments of the stochastic process $\text{a}:=a^{\text{z}}$, to a CLDS model with

$$\text{a}(\cdot )∼GP\left(\pi^{⊤}a,a^{⊤}\left(P^{\left|t_{j}-t_{i}\right|}\text{diag}(\pi )-\pi \pi^{⊤}\right)a\right),$$

over time conditions $u_{t}=t$, for a the vector of values taken by $a^{\left(z_{i}\right)}$ and π the stationary distribution of the ${\text{z}}_{t}$ discrete state process. Finally, in a similar vein, Geadah et al. (2024) consider the discrete states ${\text{z}}_{t}$ dictating the dynamics to live on a continuous support. However, they do not leverage this continuity in the parameters A(⋅) themselves.

The *recurrent SLDS* (rSLDS) model (Linderman et al., 2017) takes an important departure from the SLDS by leveraging the continuous latent states $x_{t}$ to guide the discrete state transitions. Smith et al. (2021) use this dependency but turn to the linearization of nonlinear systems, using x-space fixed points as guide for the linear dynamics. In contrast, we linearize based on observed external conditions.

### Smoothly varying dynamical systems models

Switching LDS models can be contrasted with models that smoothly interpolate between dynamical parameter regimes. The simplest example of this would be time-varying linear models (e.g. Costa et al. 2019); CLDS models are a generalization of this idea that comes with several advantages (see §2.2). Work by Costacurta et al. (2022) introduced an autoregressive model with a dynamics matrix that is subject to an approximately continuous and latent time warping factor. Unlike CLDS, this model does not infer a low-dimensional latent dynamical space. More similar to CLDS models is recent work by Hu et al. (2024). They relax the discrete switching in rSLDS models to allow smoothly varying soft mixtures of linear dynamics. Again, CLDS models achieve a similar effect but utilize observed experimental covariates to infer these dynamical transitions. Thus, CLDS models can loosely be seen as supervised analogs to these models.

## 5. Conclusion

In this work, we revisited and extended classical linear-Gaussian state space models of neural circuit dynamics. Our results suggest that these models can be competitive with modern methods when the dynamical parameters vary smoothly as a function of experimentally measured covariates. Like classical linear models, CLDS models are easy to fit and interpret. Our main technical contribution was to introduce an approximate GP prior over system parameters and show that this leads to closed form inference and parameter updates under a Gaussian noise model.

As their name implies, CLDS models assume conditionally linear latent dynamics, and this assumption brings some limitations. First, these models rely on observing a time series of experimental covariates, $u_{1:T}^{k}$. We expect performance to suffer if the covariates are corrupted for portions of time, such as during forecasting or with partial observations. Second, the model assumes linear dynamics conditioned on $u_{t}^{k}$. We believe this is a good approximation in many settings of interest, particularly when there is strong tuning to sensory or behavioral variables—i.e. when the value of $u_{t}^{k}$ can be used to accurately predict the position of $x_{t}^{k}$. We expect (see §A.2) CLDS models to struggle in other settings where external measurements are only loosely correlated with the position of $x_{t}^{k}$ (e.g. cognitive tasks with long periods of internal deliberation). In these scenarios, we expect that modern approaches that leverage deep learning (e.g. LFADS) will outperform CLDS models when given access to large amounts of data. Nevertheless, neural recordings are often trial-limited in practice (Williams & Linderman, 2021). We therefore view CLDS models as a broadly applicable modeling tool for many neuroscience applications.

Future work could extend CLDS models to overcome these limitations, such as handling partially observed covariates, $u_{t}^{k}$. Since CLDS models can be viewed as a dynamical extension of Wishart process models (see §4), future work could also apply this method to infer across-time noise correlations (reviewed in Panzeri et al., 2022), in addition to classical across-trial noise correlations. Nejatbakhsh et al. (2024) show how across-time correlations can be used to quantify similarity in dynamical systems—a topic that has recently attracted strong interest (Ostrow et al., 2023). CLDS models are a potentially attractive framework for tackling the unsolved challenge of estimating this high-dimensional correlation structure in trial-limited regimes.

## Figures and Tables

**Figure 5.**
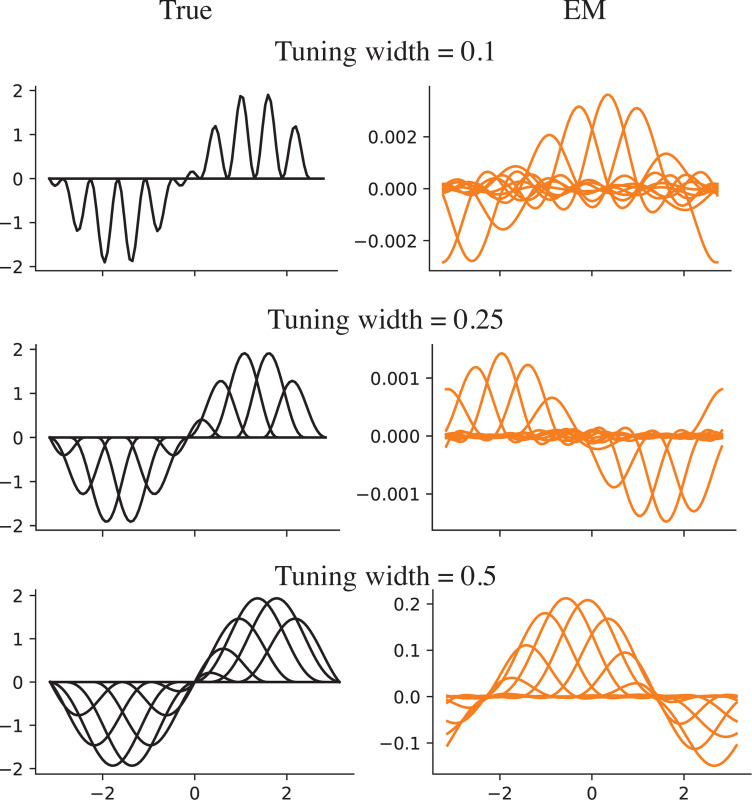
Recovery of C. Rows indicate varying level of tuning curve width γ for $C_{i,:}\left(u\right)$. Recovery becomes more challenging for smaller width since it requires a higher and higher number of bases L to approximate the true tuning bump.

**Figure 6.**
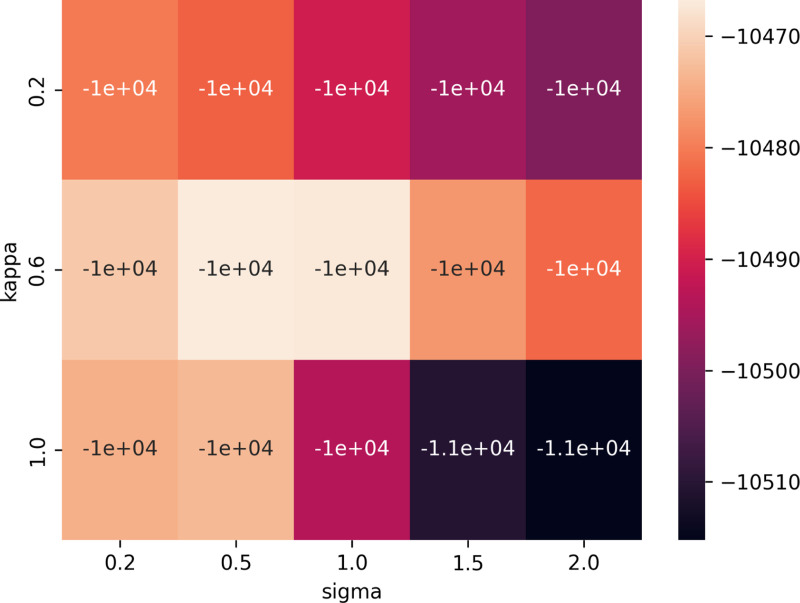
Hyperparameter search, CLDS marginal log-likelihood on a held-out validation dataset set for κ∈{0.2,0.6,1.0} and σ∈{0.2,0.5,1.0,1.5,2.0}. Maximum attained at {κ,σ}={0.6,0.5}.

## A. Modeling

### A.1. Expectation-Maximization Steps

#### A.1.1. Least squares derivation

Recall

$$M(u)X=W^{⊤}(\phi (u)⊗X),\;W\in {\mathbb{R}}^{D_{2}L\times D_{1}}$$

for $M(u)\in {\mathbb{R}}^{D_{1}\times D_{2}},\;X\in {\mathbb{R}}^{D_{2}\times M}$. In particular, $M\left(u_{n}\right)x_{n}=W^{⊤}z_{n}$. What follows are standard least-squares derivations for matrix coefficients with matrix regularization, which we include for completeness.

Our posterior objective reads as

$$\begin{array}{r} \log p\left(W|y_{1:N},x_{1:N},u_{1:N}\right)\propto \log p\left(y_{1:N}|W,x_{1:N},u_{1:N}\right)+\log p(W)\\ =\sum_{n=1}^{N}\log p\left(y_{n}|W,x_{n},u_{n}\right)+\log p(W). \end{array}$$

We have

$$\begin{array}{l} \log p\left(y_{n}|x_{n},u_{t}\right)=-\frac{1}{2}{\left(y_{n}-W^{⊤}z_{n}\right)}^{⊤}{\text{Σ}}^{-1}\left(y_{n}-W^{⊤}z_{n}\right)-c\\ \;=-\frac{1}{2}\text{Tr}\left[{\left(y_{n}-W^{⊤}z_{n}\right)}^{⊤}{\text{Σ}}^{-1}\left(y_{n}-W^{⊤}z_{n}\right)\right]-c\\ \;=-\frac{1}{2}\text{Tr}\left[{\text{Σ}}^{-1}\left(y_{n}-W^{⊤}z_{n}\right){\left(y_{n}-W^{⊤}z_{n}\right)}^{⊤}\right]-c\\ \;=-\frac{1}{2}\left(\text{Tr}\left[{\text{Σ}}^{-1}y_{n}y_{n}^{⊤}\right]-2\text{Tr}\left[{\text{Σ}}^{-1}W^{⊤}z_{n}y_{n}^{⊤}\right]+\text{Tr}\left[{\text{Σ}}^{-1}W^{⊤}z_{n}z_{n}^{⊤}W\right]\right)-c. \end{array}$$

with the normalizing constant $c=\frac{1}{2}\log|2\pi \text{Σ}|$.

To optimize this expression with respect to W, we consider the zeros of the derivative

$$\begin{array}{c} \frac{\partial }{\partial W}\log p\left(y_{n}|x_{n},u_{n}\right)=\frac{\partial }{\partial W}\text{Tr}\left[W^{⊤}z_{n}y_{n}^{⊤}{\text{Σ}}^{-1}\right]-\frac{1}{2}\frac{\partial }{\partial W}\text{Tr}\left[{\text{Σ}}^{-1}W^{⊤}z_{n}z_{n}^{⊤}W\right]\\ =z_{n}y_{n}^{⊤}{\text{Σ}}^{-1}-z_{n}z_{n}^{⊤}W{\text{Σ}}^{-1}, \end{array}$$

and

$$\log p(W)=-\frac{1}{2}∥W{∥}_{F}^{2}\;\Rightarrow \;\frac{\partial }{\partial W}\log p(W)=-W.$$

Taken together, we thus have that the stationary point of the posterior satisfies

$$\sum_{n=1}^{N}\left(z_{n}y_{n}^{⊤}{\text{Σ}}^{-1}-z_{n}z_{n}^{⊤}W{\text{Σ}}^{-1}\right)-W=0.$$

Define $Y\in {\mathbb{R}}^{N\times D_{1}}$, $Z\in {\mathbb{R}}^{N\times D_{2}L}$ by row-wise stacking $y_{n}$ and $z_{n}$ respectively, and note that $\sum_{n}z_{n}y_{n}^{⊤}=Z^{⊤}Y$. We get

$$Z^{⊤}Y{\text{Σ}}^{-1}-Z^{⊤}ZW{\text{Σ}}^{-1}-W=0\;\;\Rightarrow \;Z^{⊤}ZW+W\text{Σ}=Z^{⊤}Y$$

as desired.

#### A.1.2. Joint dynamics and bias EM update in weight-space

Here we detail how one EM update for the parameters governing {A,b} is carried out. Using the function-space weights $W_{A}\in {\mathbb{R}}^{DL\times D}$ and $W_{b}\in {\mathbb{R}}^{L\times D}$, the dynamics read

$$x_{t+1}=A\left(u_{t}\right)x_{t}+b\left(u_{t}\right)+\epsilon_{t}\;\;=W_{A}^{⊤}\underset{z_{t}}{\underset{︸}{\left(\phi \left(u_{t}\right)⊗x_{t}\right)}}+W_{b}^{⊤}\phi \left(u_{t}\right)+\epsilon_{t}$$

Define $z_{t}=\phi \left(u_{t}\right)⊗x_{t}\in {\mathbb{R}}^{LD}$, and note

$$\;E_{x_{t}}\left[z_{t}\right]=\phi \left(u_{t}\right)⊗E_{x_{t}}\left[x_{t}\right]\;E_{x_{t},x_{t+1}}\left[z_{t}x_{t+1}^{⊤}\right]=\phi \left(u_{t}\right)⊗E_{x_{t},x_{t+1}}\left[x_{t}x_{t+1}^{⊤}\right]$$

The quantity of interest for the M-step from the complete data log-likelihood is

$$E\left[\sum_{t=1}^{T-1}\log p\left(x_{t+1}|x_{t},F,u_{t}\right)\right]\;=-\frac{1}{2}E\left[\sum_{t=1}^{T-1}{\left(x_{t+1}-\left(A\left(u_{t}\right)x_{t}+b\left(u_{t}\right)\right)\right)}^{⊤}Q^{-1}\left(x_{t+1}-\left(A\left(u_{t}\right)x_{t}+b\left(u_{t}\right)\right)\right)\right]\;=-\frac{1}{2}E[\sum_{t=1}^{T-1}{\text{x}}_{t+1}^{⊤}Q^{-1}x_{t+1}-2{\text{x}}_{t+1}^{⊤}Q^{-1}A\left(u_{t}\right)x_{t}-2{\text{x}}_{t+1}^{⊤}Q^{-1}b\left(u_{t}\right)\;\;+x_{t}^{⊤}A{\left(u_{t}\right)}^{⊤}Q^{-1}A\left(u_{t}\right)x_{t}+2x_{t}^{⊤}A{\left(u_{t}\right)}^{⊤}Q^{-1}b\left(u_{t}\right)+b{\left(u_{t}\right)}^{⊤}Q^{-1}b\left(u_{t}\right)]\;=-\frac{1}{2}\text{Tr}[\sum_{t=1}^{T-1}Q^{-1}E\left[x_{t+1}x_{t+1}^{⊤}\right]-2Q^{-1}A\left(u_{t}\right)E\left[x_{t}x_{t+1}^{⊤}\right]-2Q^{-1}b\left(u_{t}\right)E\left[x_{t+1}^{⊤}\right]\;\;+A{\left(u_{t}\right)}^{⊤}Q^{-1}A\left(u_{t}\right)E\left[x_{t}x_{t}^{⊤}\right]+2A{\left(u_{t}\right)}^{⊤}Q^{-1}b\left(u_{t}\right)E\left[x_{t}^{⊤}\right]+b{\left(u_{t}\right)}^{⊤}Q^{-1}b\left(u_{t}\right)]$$

Which with the function-space weights reads as

$$L=-\frac{1}{2}\text{Tr}[\sum_{t=1}^{T-1}Q^{-1}E\left[x_{t+1}x_{t+1}^{⊤}\right]-2Q^{-1}W_{A}^{⊤}\left(\phi \left(u_{t}\right)⊗E\left[x_{t}x_{t+1}^{⊤}\right]\right)-2Q^{-1}W_{b}^{⊤}\left(\phi \left(u_{t}\right)E\left[x_{t+1}^{⊤}\right]\right)\;\;+W_{A}Q^{-1}W_{A}^{⊤}\left(\phi \left(u_{t}\right)\phi {\left(u_{t}\right)}^{⊤}⊗E\left[x_{t}x_{t}^{⊤}\right]\right)+2W_{A}Q^{-1}W_{b}^{⊤}\left(\phi \left(u_{t}\right)\phi {\left(u_{t}\right)}^{⊤}⊗E\left[x_{t}^{⊤}\right]\right)\;\;+W_{b}Q^{-1}W_{b}^{⊤}\phi \left(u_{t}\right)\phi {\left(u_{t}\right)}^{⊤}]$$

The partial derivatives satisfy, denoting $\phi_{t}=\phi \left(u_{t}\right)$,

(18)
$$\frac{\partial L}{\partial W_{A}}=\sum_{t=1}^{T-1}\left(\phi_{t}⊗E\left[x_{t}x_{t+1}^{⊤}\right]\right)Q^{-1}-\left(\phi_{t}\phi_{t}^{⊤}⊗E\left[x_{t}x_{t}^{⊤}\right]\right)W_{A}Q^{-1}-{\left(\phi_{t}\phi_{t}^{⊤}⊗E{\left[x_{t}\right]}^{⊤}\right)}^{⊤}W_{b}Q^{-1}\;\;=:N_{\text{Δ}}Q^{-1}-N_{\left(1,T-1\right)}W_{A}Q^{-1}-{\left({\text{Φ}}^{⊤}Z\right)}^{⊤}W_{b}Q^{-1}$$

(19)
$$\frac{\partial L}{\partial W_{b}}=\sum_{t=1}^{T-1}\phi_{t}E\left[x_{t+1}^{⊤}\right]Q^{-1}-\left(\phi_{t}\phi_{t}^{⊤}⊗E\left[x_{t}^{⊤}\right]\right)W_{A}Q^{-1}+\phi_{t}\phi_{t}^{⊤}W_{b}Q^{-1}-W_{b}\;\;=:{\text{Φ}}^{⊤}XQ^{-1}-{\text{Φ}}^{⊤}ZW_{A}Q^{-1}+{\text{Φ}}^{⊤}\text{Φ}W_{b}Q^{-1}$$

where we’ve defined the matrices $\text{Φ}\in {\mathbb{R}}^{(T-1)\times L}$, $Z\in {\mathbb{R}}^{(T-1)\times DL}$, $X\in {\mathbb{R}}^{(T-1)\times D}$ obtained by stacking $\phi \left(u_{t}\right)$, $\phi \left(u_{t}\right)⊗E\left[x_{t}\right]$ and $E\left[x_{t+1}\right]$ respectively for t∈{1,…,T−1}, and the sufficient statistics

$$N_{\left(t_{1},t_{2}\right)}=\sum_{t_{1}}^{t_{2}}\phi_{t}\phi_{t}^{⊤}⊗E\left[x_{t}x_{t}^{⊤}\right],\;N_{\text{Δ}}=\sum_{t=1}^{T-1}\phi \left(u_{t}\right)⊗E\left[x_{t}x_{t+1}^{⊤}\right].$$

which are all defined during our E-step. These statistics are in contrast to the “typical” sufficient stats without the weight-space parametrization

$$M_{\left(t_{1},t_{2}\right)}=\sum_{t_{1}}^{t_{2}}E\left[x_{t}x_{t}^{⊤}\right],\;M_{\text{Δ}}=\sum_{t=1}^{T-1}E\left[x_{t}x_{t+1}^{⊤}\right].$$

Incorporating the Gaussian prior on $W_{A}$ and $W_{b}$ and equating the two partial derivatives in (18–19) to 0 to obtain the stationary points, we obtain the system of equations

(20)
$$\left[\begin{array}{l} W_{A}\\ W_{b} \end{array}\right]Q+\left[\begin{array}{cc} N_{\left(1,T-1\right)} & Z^{⊤}\text{Φ}\\ {\text{Φ}}^{⊤}Z & -{\text{Φ}}^{⊤}\text{Φ} \end{array}\right]\left[\begin{array}{l} W_{A}\\ W_{b} \end{array}\right]=\left[\begin{array}{c} N_{\text{Δ}}\\ {\text{Φ}}^{⊤}X \end{array}\right]$$

which solving for $W_{A}$ and $W_{b}$ jointly amounts to solving our M-step.

### A.2. Nonlinear dynamics: linearization and composite dynamics

Consider input-driven nonlinear dynamics in $x_{t}\in {\mathbb{R}}^{D}$,

(21)
$$x_{t+1}=f\left(x_{t},u_{t}\right)+\epsilon_{t}$$

governed by $f:{\mathbb{R}}^{D}\times U\to {\mathbb{R}}^{D}$, and where $\epsilon_{t}$ is zero-mean Gaussian noise. We assume that f has continuous and bounded second-order partial derivatives in both x and u. Below we treat the state and input variables $\left(x_{t},u_{t}\right)$ as random variables that are jointly drawn from some unspecified distribution.

#### A.2.1. CLDS conditional approximation error

Let $f_{1},\ldots ,f_{D}$ denote the output dimensions of f; that is, $f\left(x_{t},u_{t}\right)={\left[\begin{array}{c} f_{1}\left(x_{t},u_{t}\right)\;\ldots \;f_{D}\left(x_{t},u_{t}\right) \end{array}\right]}^{⊤}$. In a first step to relate our CLDS dynamics to f, consider the first-order Taylor expansion for each output dimension in the first argument x about $a\in {\mathbb{R}}^{D}$. For i∈{1,…,D}, this is

(22)
$$f_{i}\left(x_{t},u_{t}\right)=f_{i}\left(a,u_{t}\right)+\nabla_{x}f_{i}{\left(a,u_{t}\right)}^{⊤}\left(x_{t}-a\right)+E_{i}$$

where $\nabla_{x}f_{i}$ denotes the vector-valued gradient of $f_{i}$ with respect to it’s first argument x and $E_{i}$ is the residual of the Taylor approximation. The Lagrange remainder form of Taylor’s theorem tells us that this residual can be expressed as:

(23)
$$E_{i}={\left(x_{t}-a\right)}^{⊤}\nabla_{x}^{2}f_{i}\left(\zeta ,u_{t}\right)\left(x_{t}-a\right)$$

for some $\zeta \in {\mathbb{R}}^{D}$, where $\nabla_{x}^{2}f_{i}$ is the matrix-valued Hessian of $f_{i}$ with respect to its first argument. We can upper bound the absolute value of this remainder using the Cauchy-Schwartz inequality and a standard operator norm inequality. Specifically, for i∈{1,…,D}, we have

(24)
$$\left|E_{i}\right|\leq {\left\|\nabla_{x}^{2}f_{i}\left(\zeta ,u_{t}\right)\right\|}_{2}{\left\|x_{t}-a\right\|}_{2}^{2}$$

where ${\left\|\nabla_{x}^{2}f_{i}\left(\zeta ,u_{t}\right)\right\|}_{2}$ denotes the maximal singular value (operator norm) of the matrix $\nabla_{x}^{2}f_{i}\left(\zeta ,u_{t}\right)\in {\mathbb{R}}^{D\times D}$. We assume that this operator norm is upper bounded globally by a constant $L_{i}>0$,

(25)
$${\left\|\nabla_{x}^{2}f_{i}(x,u)\right\|}_{2}\leq L_{i}\;\forall x,u\in {\mathbb{R}}^{D}.$$

Intuitively, this assumption implies that the second-order derivatives of f with respect to x are not too large, meaning that the accuracy of the first-order Taylor approximation degrades in proportion to the magnitude of curvature in f.

Returning to equation (24), we proceed by taking conditional expectations with respect to $x_{t}$ given $u_{t}$ on both sides of the inequality. This yields an upper bound on the expected approximation error,

(26)
$$E\left[\left|E_{i}\right||u_{t}\right]\leq L_{i}\cdot E\left[{\left\|x_{t}-a\right\|}_{2}^{2}|u_{t}\right].$$

This upper bound is minimized by choosing $a=E\left[x_{t}|u_{t}\right]$. Plugging in this choice, we observe that

(27)
$$E\left[\left|E_{i}\right||u_{t}\right]\leq L_{i}\cdot \text{Tr}\left[\mathbb{C}\text{ov}\left[x_{t}|u_{t}\right]\right]$$

where $\mathbb{C}\text{ov}\left[x_{t}|u_{t}\right]=E\left[\left(x_{t}-E\left[x_{t}|u_{t}\right]\right){\left(x_{t}-E\left[x_{t}|u_{t}\right]\right)}^{⊤}|u_{t}\right]$ is the conditional covariance of $x_{t}$ given $u_{t}$. Finally, we can sum these upper bounds over i={1,…,D} to bound the total expected approximation error as

(28)
$$\sum_{i}E\left[\left|E_{i}\right||u_{t}\right]\leq L\cdot \text{Tr}\left[\mathbb{C}\text{ov}\left[x_{t}|u_{t}\right]\right]$$

where we have defined $L=\sum_{i}\;L_{i}$ as a global constant bounding the second-order smoothness of f across all dimensions. Returning to equation (22) and plugging in the optimal choice of $a=E\left[x_{t}|u_{t}\right]$, we obtain the following CLDS approximation to the nonlinear dynamics

(29)
$$h\left(x_{t},u_{t}\right):=f\left(E\left[x_{t}|u_{t}\right],u_{t}\right)+\nabla_{x}f\left(E\left[x_{t}|u_{t}\right],u_{t}\right)\left(x_{t}-E\left[x_{t}|u_{t}\right]\right)=A\left(u_{t}\right)x_{t}+b\left(u_{t}\right)$$

where $\nabla_{x}f$ is the matrix-valued Jacobian, $\nabla_{x}f(x,u)\in {\mathbb{R}}^{D\times D}$, with respect to the first argument of f and we have re-arranged the terms and defined

(30)
$$A\left(u_{t}\right):=\nabla_{x}f\left(E\left[x_{t}|u_{t}\right],u_{t}\right),\;b\left(u_{t}\right):=f\left(E\left[x_{t}|u_{t}\right],u_{t}\right)-\nabla_{x}f\left(E\left[x_{t}|u_{t}\right],u_{t}\right)E\left[x_{t}|u_{t}\right].$$

For each value of $u_{t}$, the quality of this approximation is guaranteed by equation (28) to be small if the second-order derivatives of f with respect to x are small and if the conditional variance of $x_{t}$ given $u_{t}$ is small. We note that this analysis of approximation error and does not account for the additional estimation error we incur when learning the functions A(u) and b(u) from noisy and limited data. Nonetheless, this analysis tells us that we expect the CLDS to perform well in circumstances where the underlying dynamics are smooth and the conditional distributions of $x_{t}$ given $u_{t}$ have low variance.

#### A.2.2. Composite dynamics

When considering the *composite dynamics* in (10) (Section §3.1), we are interested in approximating the input-driven nonlinear dynamics given by equation (21) with autonomous nonlinear dynamics, governed by some function g such that

(31)
$$f\left(x_{t},u_{t}\right)\approx g\left(x_{t}\right).$$

We can evaluate the quality of this approximation using the conditional expectation of the squared error

(32)
$$E\left[{\left\|f\left(x_{t},u_{t}\right)-g\left(x_{t}\right)\right\|}_{2}^{2}|x_{t}\right]$$

where the conditional expectation is taken over $u_{t}$ given $x_{t}$. This approximation error is minimized by choosing

(33)
$$g\left(x_{t}\right)=E\left[f\left(x_{t},u_{t}\right)|x_{t}\right]=E\left[x_{t+1}|x_{t}\right]$$

However, learning f from limited data is challenging, so we replace this with our CLDS model to achieve the composite dynamical system

(34)
$$g\left(x_{t}\right)\approx E\left[h\left(x_{t},u_{t}\right)|x_{t}\right]$$

where h(x,u)=A(u)x+b(u) as in equation (29). Now we analyze the quality of this approximation. Consider the residual along dimension i∈{1,…,D},

(35)
$$R_{i}=f_{i}\left(x_{t},u_{t}\right)-E\left[h_{i}\left(x_{t},{\tilde{u}}_{t}\right)|x_{t}\right]$$

where the expectation in the second term is taken over ${\tilde{u}}_{t}$, which is drawn from the conditional distribution of $u_{t}$ given $x_{t}$. That is, $R_{i}$ is a random variable that depends on a joint sample of $\left(x_{t},u_{t}\right)$ from the stationary distribution, and ${\tilde{u}}_{t}$ is a dummy variable that is integrated out during the calculation of $R_{i}$. To proceed, we note that

(36)
$$R_{i}=E\left[f_{i}\left(x_{t},u_{t}\right)-h_{i}\left(x_{t},{\tilde{u}}_{t}\right)|x_{t}\right]$$

(37)
$$=E\left[f_{i}\left(x_{t},u_{t}\right)-f_{i}\left(x_{t},{\tilde{u}}_{t}\right)+f_{i}\left(x_{t},{\tilde{u}}_{t}\right)-h_{i}\left(x_{t},{\tilde{u}}_{t}\right)|x_{t}\right]$$

and, applying Jensen’s inequality and the triangle inequality, we conclude

(38)
$$\left|R_{i}\right|\leq E_{{\tilde{u}}_{t}|x_{t}}\left[\left|f_{i}\left(x_{t},u_{t}\right)-f_{i}\left(x_{t},{\tilde{u}}_{t}\right)+f_{i}\left(x_{t},{\tilde{u}}_{t}\right)-h_{i}\left(x_{t},{\tilde{u}}_{t}\right)\right|\right]$$

(39)
$$\leq E_{{\tilde{u}}_{t}|x_{t}}\left[\left|f_{i}\left(x_{t},u_{t}\right)-f_{i}\left(x_{t},{\tilde{u}}_{t}\right)\right|\right]+E_{{\tilde{u}}_{t}|x_{t}}\left[\left|f_{i}\left(x_{t},{\tilde{u}}_{t}\right)-h_{i}\left(x_{t},{\tilde{u}}_{t}\right)\right|\right]$$

where we have introduced a minor change in notation, using $E_{{\tilde{u}}_{t}|x_{t}}$ to denote the conditional expectation of ${\tilde{u}}_{t}$, given $x_{t}$. Now we take expectations on both sides of this inequality with respect to the remaining random variables, $x_{t}$ and $u_{t}$, which are sampled from some stationary distribution associated with the dynamical system. Using the law of total expectation, we obtain

(40)
$$E\left|R_{i}\right|\leq E_{x_{t}}\left[E_{u_{t},{\tilde{u}}_{t}|x_{t}}\left[\left|f_{i}\left(x_{t},u_{t}\right)-f_{i}\left(x_{t},{\tilde{u}}_{t}\right)\right|\right]\right]+E_{u_{t}}\left[E_{x_{t}|u_{t}}\left[\left|f_{i}\left(x_{t},u_{t}\right)-h_{i}\left(x_{t},u_{t}\right)\right|\right]\right]$$

On the right hand side, the first term takes the conditional expectation over $u_{t}$ and ${\tilde{u}}_{t}$, followed by an expectation over $x_{t}$. The second term reverses this order, taking the conditional expectation over $x_{t}$, followed by an expectation over $u_{t}$ (since this is identically distributed to ${\tilde{u}}_{t}$, we drop the tilde).

To upper bound the first term, we introduce an assumption that $f_{i}$ is Lipschitz continuous in its second argument. That is, there exists a constant $C_{i}>0$ such that

(41)
$$\left|f_{i}\left(x_{t},u\right)-f_{i}\left(x_{t},u^{\prime }\right)\right|\leq C_{i}{\left\|u-u^{\prime }\right\|}_{2}\;\forall u,u^{\prime }\in U.$$

In conjunction with Jensen’s inequality, this Lipschitz assumption implies the following upper bound:

(42)
$$E_{u_{t},{\tilde{u}}_{t}|x_{t}}\left[\left|f_{i}\left(x_{t},u_{t}\right)-f_{i}\left(x_{t},{\tilde{u}}_{t}\right)\right|\right]\leq C_{i}\cdot E_{u_{t},{\tilde{u}}_{t}|x_{t}}\left[{\left\|u_{t}-{\tilde{u}}_{t}\right\|}_{2}\right]$$

(43)
$$\leq C_{i}\sqrt{E_{u_{t},{\tilde{u}}_{t}|x_{t}}{\left\|u_{t}-{\tilde{u}}_{t}\right\|}_{2}^{2}}$$

(44)
$$=C_{i}\sqrt{2\text{Tr}\left[\mathbb{C}\text{ov}\left[u_{t}|x_{t}\right]\right]}$$

It remains to upper bound the second term in equation (40). A direct application of the results in §A.2.1 yields the bound

(45)
$$E_{x_{t}|u_{t}}\left[\left|f_{i}\left(x_{t},u_{t}\right)-h_{i}\left(x_{t},u_{t}\right)\right|\right]\leq L_{i}\cdot \text{Tr}\left[\mathbb{C}\text{ov}\left[x_{t}|u_{t}\right]\right]$$

where $L_{i}$, defined in (25), is a constant bounding the second derivatives of $f_{i}$ with respect to $x_{t}$. Putting these pieces together we conclude that

(46)
$$E\left|R_{i}\right|\leq C_{i}\cdot E_{x_{t}}\sqrt{2\text{Tr}\left[\mathbb{C}\text{ov}\left[u_{t}|x_{t}\right]\right]}+L_{i}\cdot E_{u_{t}}\text{Tr}\left[\mathbb{C}\text{ov}\left[x_{t}|u_{t}\right]\right]$$

And so an upper bound on the total absolute error of the composite dynamics is given by

(47)
$$\sum_{i=1}^{D}E\left|R_{i}\right|\leq C\cdot E_{x_{t}}\sqrt{2\text{Tr}\left[\mathbb{C}\text{ov}\left[u_{t}|x_{t}\right]\right]}+L\cdot E_{u_{t}}\text{Tr}\left[\mathbb{C}\text{ov}\left[x_{t}|u_{t}\right]\right]$$

where we have defined $C=\sum_{i}C_{i}$ and $L=\sum_{i}L_{i}$.

In summary, we have shown that the approximation error of the composite dynamical system, defined in equation (10), is bounded by a sum of two terms. The first term approaches zero in the limit that the conditional covariance of $u_{t}$ given $x_{t}$ goes to zero, while the second term approaches zero in the limit that the conditional covariance of $x_{t}$ given $u_{t}$ goes to zero. Thus, the composite dynamics have the potential to provide an accurate depiction of the true nonlinear dynamical system if $x_{t}$ and $u_{t}$ are close to being in one-to-one correspondence with each other.

We note that in practice when computing the composite dynamics in (10), we make the simplifying assumption that $p\left(u_{t}|x_{t}=x\right)$ does not depend on t (i.e. we assume that this decoding distribution is stationary).

### A.3. Correspondence between CLDS and Switching LDS

In this section, we explore the relationship between the linear time-variant dynamics of (1a) with $A_{ij}\overset{iid}{∼}GP\left(0,k_{t}\right)$, and the dynamics of a switching linear dynamical system. While the latter has parameters evolving over a discrete set, we can nonetheless explore how to think of this discrete support as embedded within ${\mathbb{R}}^{D\times D}$, and seek to match the moments of these two processes.

The first thing to note is that by drawing the entries of $A_{ij}$ i.i.d., we can gain insight by considering a single process $\text{a}(\cdot )∼GP\left(0,k_{t}\right)$, and a SLDS with one-dimensional dynamics. Thus, we consider the SLDS model

(48a)
$$p\left({\text{z}}_{t+1}=i|{\text{z}}_{t}=j\right)=P_{ij}$$

(48b)
$${\text{x}}_{t+1}={\text{a}}^{\left({\text{z}}_{t}\right)}{\text{x}}_{t}+\epsilon_{t}$$

with discrete states ${\text{z}}_{t}$ governing dynamics in ${\text{x}}_{t}$, with transition matrix p and dynamics ${\text{a}}^{\left(z\right)}$ for z∈Z. Denote $z={\left(z_{1},\ldots ,z_{K}\right)}^{⊤}\in {\mathbb{R}}^{\left|Z\right|}$ for the vector of the |Z|=K values that can be taken by the stochastic process z, and similarly $a={\left(a^{\left(z_{1}\right)},\ldots ,a^{\left(z_{K}\right)}\right)}^{⊤}$. We consider π the stationary distribution for the z process. Finally, note that the map $z_{n}\to a^{\left(z_{n}\right)}$ is deterministic, one-to-one and onto, such that we can think of the Markov chain in ${\text{z}}_{t}$ as having support over a. Let ${\text{a}}_{t}:=a^{\left({\text{z}}_{t}\right)}$, and denote $a_{i}=a^{\left(z_{i}\right)}$.

Let us now explore the moments of the stochastic process ${\text{a}}_{t}$ to determine its relationship with the CLDS. Assume ${\text{z}}_{t}$ has reached stationarity, such that $p\left({\text{z}}_{t}\right)=p\left({\text{a}}_{t}\right)=\pi$. Then, first,

(49)
$$E[\text{a}]=\pi^{⊤}a$$

and then we have the cross-correlation

(50)
$$\begin{array}{l} E\left[{\text{a}}_{t}{\text{a}}_{t+n}\right]=E\left[E\left[{\text{a}}_{t}{\text{a}}_{t+n}|{\text{a}}_{t}\right]\right]\\ \;=\sum_{j=1}^{K}E\left[{\text{a}}_{t}{\text{a}}_{t+n}|{\text{a}}_{t}=a_{j}\right]p\left({\text{a}}_{t}=a_{j}\right)\\ \;=\sum_{j=1}^{K}\sum_{i=1}^{K}a_{j}\left(p\left({\text{a}}_{t+n}=z_{i}|{\text{a}}_{t}=a_{j}\right)\right)p\left({\text{a}}_{t}=a_{j}\right)\\ \;=\sum_{i=1}^{K}\sum_{j=1}^{K}a_{i}a_{j}P_{ij}^{n}\pi_{j}\\ \;=a^{⊤}P^{n}\text{diag}(\pi )a. \end{array}$$

Denote Π=diag(π). We get the desired covariance

(51)
$$\mathrm{cov}\left({\text{a}}_{t},{\text{a}}_{t+n}\right)=E\left[{\text{a}}_{t}{\text{a}}_{t+n}\right]-E\left[{\text{a}}_{t}\right]E\left[{\text{a}}_{t+n}\right]=a^{⊤}\left(P^{n}\text{Π}-\pi \pi^{⊤}\right)a$$

yielding our kernel form

(52)
$$\mathrm{cov}\left(\text{a}\left(t_{i}\right),\text{a}\left(t_{j}\right)\right)=k_{t}\left(t_{i},t_{j}\right)=a^{⊤}\left(P^{\left|t_{j}-t_{i}\right|}\text{Π}-\pi \pi^{⊤}\right)a$$

Hence, in all, our approximation of the stochastic process ${\text{a}}_{t}$ over ℝ up to the first two moments is

(53)
$$\text{a}(\cdot )∼GP\left(\pi^{⊤}a,a^{⊤}\left(P^{\left|t_{j}-t_{i}\right|}\text{Π}-\pi \pi^{⊤}\right)a\right)$$

which in particular can be made zero-mean by consider values a such that $\pi^{⊤}a=0$. This establishes the form of the GP prior over a(⋅) for which the corresponding CLDS best matches the SLDS.

## B. Experiments

### B.1. Task-agnostic model implementations

We initialize observation matrices for all models (C in (C)LDS models, log-rate decoder weights in LFADS) as the PCA principal axes in y-space—that is, the top D right singular vectors of the data—for each task.

#### CLDS

In all experiments, we assume that $d\left(u_{t}\right)=0$, which forces the predicted firing rates, conditioned on $u_{t}$, to lie in a D-dimensional space spanned by the columns of $C\left(u_{t}\right)$.

#### LFADS

We use the Jax implementation of LFADS available at https://github.com/google-research/computation-thru-dynamics/tree/master/lfads_tutorial. We choose the factor dimension to be the same as the latent dimension D of the (C)LDS models and the inferred inputs to be of dimension |U|, both following the (C)LDS models on any given experiment. The other components of the architecture are held fixed across all experiments:

- Encoder, controller and generator have hidden-states of dimension 32;
- The inferred inputs are modeled as having an auto-correlation of 1.0 and a variance of 0.1;
- We train for 1000 epochs, with an initial learning rate of 0.5 with exponential decay at rate 0.995, along with a KL warm-up coming in at 500 steps.

### B.2. Synthetic experiment and parameter recovery

For the peaks $\xi_{i}$ spanning regularly the interval [−π,π) and widths γ, the tuning curves in the rows of C for the synthetic experiment are defined as

(54)
$$C_{i,:}(u)=\{\begin{array}{ll} \left(1+\cos\left(\frac{u-\xi_{i}}{\gamma }\right)\right)u^{⊤} & \text{if}\;u\in \left(\xi_{i}-\gamma \pi ,\xi_{i}+\gamma \pi \right)\\ 0 & \text{else} \end{array}$$

We plot the its recovery with our inference procedure in Fig. 5, up to the invertible transform non-identifiability inherent to LDS models.

### B.3. Pre-processing

#### Mice Head-Direction

We considered neural activity from the “wake” period, binned in 50ms time-bins, then processed to firing rates and separated into 10s trials.

#### Macaque center-out reaching

We analyzed neural recordings of dorsal premotor cortex (PMd) in macaques performing center-out reaching task from N. Even-Chen, B. Sheffer et al. (2019). The experiments supported 3 different reach radii, and we selected only the middle reach radius at 8cm. We were left with only the angular direction as the reach condition, over N=16 possible reach angles. We aligned all trials around the go-cue, selecting 200 ms before the go-cue and 300ms after. We binned the data into 5ms bins, and performed Gaussian kernel smoothing with a standard deviation of 0.5 over bins.

### B.4. Hyper-parameters

Throughout all experiments, we’ve set L=5 to balance expressivity and number of parameters. To select the other hyper-parameters of GP prior length-scale κ and scale σ, we evaluated the various models on a held-out validation set. We plot in Fig. 6 our search over {κ,σ} on the macaque center-out reaching data.
